# Supplementary material for: Causal Relationship between Aging and Anorexia Nervosa: A White-Matter-Microstructure-Mediated Mendelian Randomization Analysis
Source: Biomedicines. 2024 Aug 16;12(8):1874. doi: 10.3390/biomedicines12081874 (PMC11351342; doi:10.3390/biomedicines12081874)
Supplement: Supplementary file 1 [file biomedicines-12-01874-s001.zip › Supplementary Figure/Figure S4/Singe SNP plot of MR analysis for TL to ubm-b-1492.pdf]

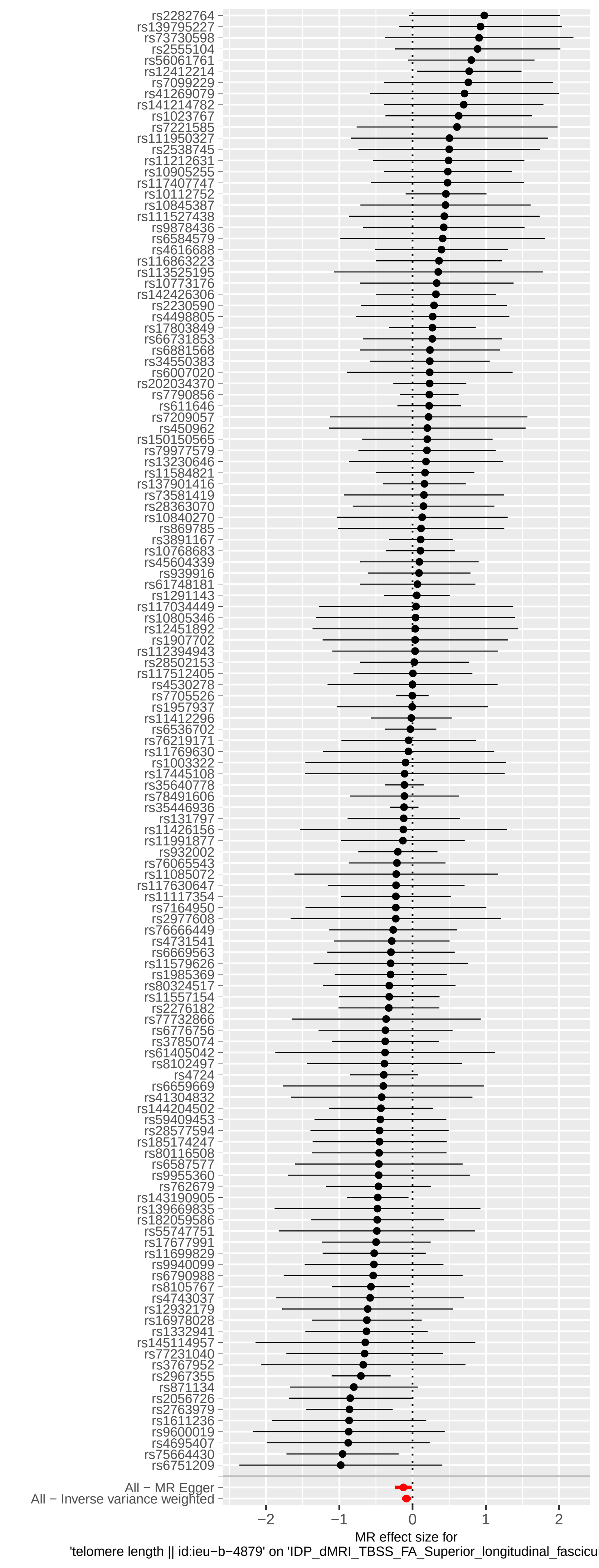
rs2282764
rs139795227
rs73730598
rs2555104
rs56061761
rs12412214
rs7099229
rs41269079
rs141214782
rs1023767
rs7221585
rs111950327
rs2538745
rs11212631
rs10905255
rs117407747
rs10112752
rs10845387
rs111527438
rs9878436
rs6584579
rs4616688
rs116863223
rs113525195
rs10773176
rs142426306
rs2230590
rs4498805
rs17803849
rs66731853
rs6881568
rs34550383
rs6007020
rs202034370
rs7790856
rs611646
rs7209057
rs450962
rs150150565
rs79977579
rs13230646
rs11584821
rs137901416
rs73581419
rs28363070
rs10840270
rs869785
rs3891167
rs10768683
rs45604339
rs939916
rs61748181
rs1291143
rs117034449
rs10805346
rs12451892
rs1907702
rs112394943
rs28502153
rs117512405
rs4530278
rs7705526
rs1957937
rs11412296
rs6536702
rs76219171
rs11769630
rs1003322
rs17445108
rs35640778
rs78491606
rs35446936
rs131797
rs11426156
rs11991877
rs932002
rs76065543
rs11085072
rs117630647
rs11117354
rs7164950
rs2977608
rs76666449
rs4731541
rs6669563
rs11579626
rs1985369
rs80324517
rs11557154
rs2276182
rs77732866
rs6776756
rs3785074
rs61405042
rs8102497
rs4724
rs6659669
rs41304832
rs144204502
rs59409453
rs28577594
rs185174247
rs80116508
rs6587577
rs9955360
rs762679
rs143190905
rs139669835
rs182059586
rs55747751
rs17677991
rs11699829
rs9940099
rs6790988
rs8105767
rs4743037
rs12932179
rs16978028
rs1332941
rs145114957
rs77231040
rs3767952
rs2967355
rs871134
rs2056726
rs2763979
rs1611236
rs9600019
rs4695407
rs75664430
rs6751209
All − MR Egger
All − Inverse variance weighted
−2
−1
0
1
2
MR effect size for
'telomere length || id:ieu−b−4879' on 'IDP_dMRI_TBSS_FA_Superior_longitudinal_fascicul
